# Supplementary material for: Patterns of Intron Gain and Loss in Fungi
Source: PLoS Biol. 2004 Nov 30;2(12):e422. doi: 10.1371/journal.pbio.0020422 (PMC532390; doi:10.1371/journal.pbio.0020422)
Supplement: Table S1 — Also available at http://genes.mit.edu/NielsenEtAl/. (4.3 MB ZIP). [file pbio.0020422.st001.zip › NielsenEtAl/html/898.html]

AN7230.1.NCU00206.1.MG11036.1.FG09085.1


```
 CLUSTAL W (1.82) Multiple Sequence Alignments - Introns Inserted


Sequence 1: NCU00206.1	829 aa
Sequence 2: MG11036.1	840 aa
Sequence 3: FG09085.1	797 aa
Sequence 4: AN7230.1	780 aa
Alignment Length: 859 aa
Number Identitical Residues: 288 aa
Alignment Score (without introns) 17243


MG11036.1 	MRTLAPFIATLLVAVTY1LQPSLAQGPLPQVYTD~PETGIVFNTWSATDGPIQGEGYGSV
NCU00206.1	MRTTSAFLSGLAAVASL1LSPAFAQ-TAPKTFTH~PDTGIVFNTWSASD---------SQ
FG09085.1 	-----------------~----------------~-------------------------
AN7230.1  	MHSFLRSFAALVAAGS-~---------------D1PDTGIVFDTWTVEA---------SS
          	  :     :      :                   ..:.   .: :            : 

MG11036.1 	SLGGFTFG~MALPSDAL~TVDANEFIGYL0ECSTPTT-PGEGWCGVSMGGSMTRALLLVA
NCU00206.1	TKGGFTVG~MALPSNAL~TTDATEFIGYL0ECSSAKNGANSGWCGVSLRGAMTNNLLITA
FG09085.1 	MASGHSIS2LTTFTSHS0TTNADEFIGYL0NCATPKG-KSTGWCGLSYGGSMTNSLLLFA
AN7230.1  	SSAGFTFG~VSLPEDAL~DTDATEFIGYL0SCSSSSTSEFTGWCGLSMGSSMNSNLLLVA
          	  .*.:.. ::   .    .:* ****** .*::.. .   ****:*  .:*.  **: *

MG11036.1 	WPHEDEVLTSFRYATGFVVPELYKGDAKLTQITSSMNSTHYKLVFRCQNCLKWSHDGLEG
NCU00206.1	WPSDGEVYTNLMFATGYAMPKNYAGDAKITQIASSVNATHFTLVFRCQNCLSWDQDGVTG
FG09085.1 	YPHDGEILTTFHWASAQVEPVQYKGKATLTQISSKITDDGFTLIYRCQGCTSWSQDGSSG
AN7230.1  	YAQDDTVLTSFRFSSGYAMPSVYSGNATLTQISSTVTADKFEVLFRCEECLRWDHEGVSG
          	:. :. : *.: :::. . *  * *.*.:***:*.:.   : :::**: *  *.::*  *

MG11036.1 	GQSTSSSFLMLGWGQARTPVGNPECPDRVVIQQHDNGMNLFPAPLDVSIANPSYASWAAT
NCU00206.1	GISTSNKGAQLGWVQAFPSPGNPTCPTQITLSQHDNGMGQWGAAFDSNIANPSYTAWAAK
FG09085.1 	RLATTSTVAVLGWAHSLTNAINPECPAEARMLQHET-QSIFGGQLTSNAFNSKYADWAKL
AN7230.1  	SATTSAGQLILAWAQAEESPTNADCPDDLSLVQHEA-QGIWVGKLSGDAATSNYETWAAL
          	  :*:     *.* ::     *. **    : **:   . : . :  .  ...*  **  

MG11036.1 	ATKEVTGDCGGST--------EPPVPEVTGVPVPSGT-YDYVVVGAGAGGIPIADKLSEA
NCU00206.1	ATKTVTGTCSG-----------PVTTSIAATPVPTGVSFDYIVVGGGAGGIPVADKLSES
FG09085.1 	ATKVVKGDCDGSEPTATATGEPTPTAEPTGNPVPNET-YDYVVVGGGAGGLPIADRLSEA
AN7230.1  	ATNVVDGTCGTDG----GGGGDNGNGTTPGVPVPTNVTYDYIIVGSGPAGMVLADRLSEA
          	**: * * *. .     . .        .. ***. .::**::**.*..*: :**:***:

MG11036.1 	GKSVLLIEKGPPSTGRWGGNLKPSWLQGTDLTRFDVPGLCNQIWVDSNGIACDDTDQMAG
NCU00206.1	GKSVLLIEKGFASTGEHGGTLKPEWLNNTSLTRFDVPGLCNQIWKDSDGIACSDTDQMAG
FG09085.1 	GKKVLLIEKGPPSTGRWGGTRKPDWLKGTDLTRFDVPGLCNQLWVDSAGIACPDTDQMAG
AN7230.1  	GAKTLLIEKGPPSIGLWNGTMKPDWLNGTDLTRFDVPGLCNEIWKNSDGIACPDNDQMAG
          	* ..****** .* *  .*. **.**:.*.***********::* :* **** *.*****

MG11036.1 	CVLGGGTAVNAGLWWK0PYTPDWDQVFPSGWKASDMAGATSRVFNRIPGTETPSQDGKRY
NCU00206.1	CVLGGGTAINAGLWYK~PYTKDWDYLFPSGWKGSDIAGATSRALSRIPGTTTPSQDGKRY
FG09085.1 	CVLGGGTAVNAGLWWR~PTPEDWDYNFPDGWKAKDMAKYEQRVFSRIPGTMVPSMDGKLY
AN7230.1  	CLVGGGTAVNSGLWWK~PYSKDFDESFPETWKYDDVRDAVTRVFTRIPGTTTPSTDNRLY
          	*::*****:*:***:: * . *:*  **. ** .*:     *.:.***** .** *.: *

MG11036.1 	RTEGLNTLMGGLKSAGWAEVSSLREPDRKNRTVMPGPFMFANGERGGPLATYLDTASKRS
NCU00206.1	LQQGFEVLANGLKASGWKEVDSLKDSEQKNRTFSHTSYMYINGERGGPLATYLVSAKKRS
FG09085.1 	LDQGFKVVAGALNKAGWGEVNALKEPTAKNHTYTHPPFMFSGGERGGPLATYLVSASKRN
AN7230.1  	LAEGPSVIMNGLLASGWKGTTFNDEPEEKYKSVGYSPYMFSHGQRNGPMATYLLDAYQRP
          	  :* ..: ..*  :**  .    :.  * ::    .:*:  *:*.**:****  * :* 

MG11036.1 	NFKLWMNTAVKRVVREGGHVTGLEVEPFRNGGYVGTVQLTPGTGRVILSAGTFGSAKLLM
NCU00206.1	NFKLWLNTAVKRVIREGGHITGVEVEAFRNGGYSGIIPVTNTTGRVVLSAGTFGSAKILL
FG09085.1 	NFKLWTNTQVRRLVRSGGHVTGLEVEPFAGSGYAGTVKLTPETGRVILAAGTFGSAKILL
AN7230.1  	NFDLWVNTVVRRVVRDGATVTGVEVEPFNDGGYEGSLQLN-EGGRVILSAGAFGTPKILF
          	**.** ** *:*::*.*. :**:***.* ..** * : :.   ***:*:**:**:.*:*:

MG11036.1 	RS~GIGRKDQLEIV-KGSRDGPTMVDEASWIPLPVGDNLVDHVN0TDTVVSHPNVVFYDF
NCU00206.1	RS~GIGPKDQLEVV-KASADGPTMVSNSSWIDLPVGHNLVDHTN~TDTVIQHNNVTFYDF
FG09085.1 	RS1GIGPEDQLDIVSKSSRDGSSMIKKDQWINLPVGYNLEDHTN0TDAVIKHPDVVFYDF
AN7230.1  	RS1GIGPEDQLAIVNGSASDGETMISEDQWINLPVGENLMDHPN0TEIVVQHPDVVFYDY
          	** *** :*** :*. .: ** :*:.: .** **** ** ** * *: *:.* :*.***:

MG11036.1 	YEAWTSPNPADKDAYLS1KRSGILAQAAPNIGPL2FFDQIRGADGKTRHIQWTARVEGSL
NCU00206.1	YKAWDNPNTTDMNLYLN~GRSGIFAQAAPNIGPL~FWEEITGADGIVRQLHWTARVEGSF
FG09085.1 	YEAWDTPNAGDKSKYLD1ARSGILAQAAPNIGPI~IFDEIRGADNRVRSMQWTARVEASL
AN7230.1  	YAAYDDPIEADAQSYLV1NRTGPLAQSAPNVNPV~FFDQVTGSDNVTRQLQYQARVEGSH
          	* *:  *   * . **   *:* :**:***:.*: ::::: *:*. .* ::: ****.* 

MG11036.1 	GAPNGK1TMTMSQYLGTGSTSRGQMSITSGLSTVVSTHPYLHTEGDKAAVIQGIVNIQNA
NCU00206.1	ETPDGY~AMTMSQYLGRGATSRGRMTLSPTLNTVVSDLPYLKDPNDKAAVVQGIVNLQKA
FG09085.1 	GENDTK1AMTLSQYLGRGAKSRGRMTITSALNTVVSGNPFLNDPEDKKAVVKALDNLRKA
AN7230.1  	NVADGH~TISISQYVGRGQTSRGKLTITSALNTVVSTLPWLQDDNDTDAVIAGLERLRDS
          	   :   ::::***:* * .***:::::. *.****  *:*:   *. **: .: .::.:

MG11036.1 	LKGVTGLKFEFPNATTTAADYVAS0MPVSPSNRRANHWMGTAKLGTDDGRQSGGTAVVDT
NCU00206.1	LANVKGLTWAYPSANQTAADFVDK0QPVTYQSRRSNHWMGTNKMGTDDGR-SGGTAVVDT
FG09085.1 	LSGVKDLVWLQPAPSVSSQQYVDD0MVVSWSNRRANHWIGTNKMGKDDGR-QGGTAVVDL
AN7230.1  	LSTIQGLTWAYPKANVSMAEHVNS~MAKT--GRGSNHWMGSCKMGPDDGR-DGGSSVVDL
          	*  : .* :  * .. :  :.* .    :  .* :***:*: *:* **** .**::*** 

MG11036.1 	NTRVFGTDNLFVVDASIFPGMVTTNPSAYIVVASERAAERILALPPSTPLPRYSQCGGRA
NCU00206.1	NTRVYGTDNLYVVDASIFPGVPTTNPTAYIVVAAEHAAAKILAQPANEAVPKWGWCGGPT
FG09085.1 	NTKVYGTDNLFITDASIFPGMVTTNPSAYFVTASEKASDAILALPVSKAQEPYKQCGGTE
AN7230.1  	NTKVYGMDNLFVVDASIFPGMISTNPSAYITVVAERAAERILALQG--------------
          	**:*:* ***::.*******: :***:**:...:*:*:  ***                 

MG11036.1 	HTGSFQCAAPYTCQVQNEYYSQ0P----~----
NCU00206.1	YTGSQTCQAPYKCEKQNDWYWQ~CV---~----
FG09085.1 	WNGSMQCGKGYYCKALNPFYGQ~CYPEV1SYIG
AN7230.1  	----------------------~-----~----
          	
```
